# The severity of behavioural symptoms in FTD is linked to the loss of GABRQ‐expressing VENs and pyramidal neurons

**DOI:** 10.1111/nan.12798

**Published:** 2022-02-24

**Authors:** Priya Gami‐Patel, Marta Scarioni, Femke H. Bouwman, Baayla D. C. Boon, John C. van Swieten, Netherlands Brain Bank, Annemieke J. M. Rozemuller, August B. Smit, Yolande A. L. Pijnenburg, Jeroen J. M. Hoozemans, Anke A. Dijkstra

**Affiliations:** ^1^ Department of Pathology, Amsterdam Neuroscience Amsterdam University Medical Centre Amsterdam Netherlands; ^2^ Department of Neurology, Alzheimer Centre, Amsterdam Neuroscience Amsterdam University Medical Centre Amsterdam Netherlands; ^3^ Department of Neurology, Alzheimer Centre Erasmus MC Rotterdam Netherlands; ^4^ Netherlands Brain Bank Netherlands Institute for Neuroscience Amsterdam Netherlands; ^5^ Department of Molecular and Cellular Neurobiology, Centre for Neurogenomics and Cognitive Research, Amsterdam Neuroscience VU University Amsterdam Amsterdam Netherlands

**Keywords:** frontotemporal dementia (FTD), GABRQ, social–emotional behaviour, von Economo neuron (VEN)

## Abstract

**Aims:**

The loss of von Economo neurons (VENs) and GABA receptor subunit theta (GABRQ) containing neurons is linked to early changes in social–emotional cognition and is seen in frontotemporal dementia (FTD) due to *C9orf72* repeat expansion. We investigate the vulnerability of VENs and GABRQ‐expressing neurons in sporadic and genetic forms of FTD with different underlying molecular pathology and their association with the presence and severity of behavioural symptoms.

**Methods:**

We quantified VENs and GABRQ‐immunopositive neurons in the anterior cingulate cortex (ACC) in FTD with underlying TDP43 (FTLD‐TDP) (*n* = 34), tau (FTLD‐tau) (*n* = 24) or FUS (FTLD‐FUS) (*n* = 8) pathology, neurologically healthy controls (*n* = 12) and Alzheimer's disease (AD) (*n* = 7). Second, we quantified VENs and the GABRQ‐expressing population in relation to presence of behavioural symptoms in the first years of disease onset.

**Results:**

The number of VENs and GABRQ‐expressing neurons and the ratio of VENs and GABRQ‐expressing neurons over total Layer 5 neuronal population decreased in FTLD‐TDP and FTLD‐FUS, but not in FTLD‐tau, compared to control and AD. The severity of early behavioural symptoms in all donors correlated with a lower VEN and GABRQ neuronal count.

**Conclusion:**

We show that in FTD, a loss of VENs together with GABRQ‐expressing pyramidal neurons is associated with TDP43 and FUS pathology. No significant loss was found in donors with FTLD‐tau pathology; however, this could be due to the specific MAPT mutation studied and small sporadic FTLD‐tau sample size. Overall, we show the GABRQ‐expressing population correlates with behavioural changes and suggest they are key in modulating behaviour in FTD.

## Graphical abstract

The GABA receptor subunit theta (GABRQ)‐expressing neurons include the von Economo neurons (VENs), of which selective vulnerability has been linked to FTD in the earliest stages of the disease. In this study, we show there is selective loss of GABRQ‐expressing neurons in the anterior cingulate cortex in FTD with underlying TDP‐43 and FUS pathologies but not with tau pathology. Additionally, we find the selective loss of GABRQ‐expressing neurons to be associated with the behavioural symptom severity seen in FTD.

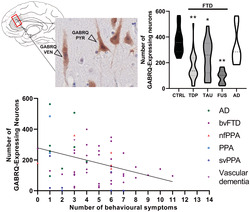

**Key Points**

- Loss of VENs and GABRQ‐expressing neurons in the ACC is associated with behavioural symptom severity in FTD.
- Loss of VENs and GABRQ‐expressing neurons in the ACC is observed in donors with FTLD‐TDP and FTLD‐FUS molecular subclasses.
- No significant loss of VENs and GABRQ‐expressing neurons was found in donors with FTLD‐tau pathology.

## INTRODUCTION

Behavioural changes are one of the most prominent clinical symptoms in frontotemporal dementia (FTD). The majority of FTD patients develop one or multiple behavioural symptoms during their disease course, of which the early unifying core feature is a lack of social and emotional cognition. Symptoms include behavioural disinhibition, apathy or inertia, loss of empathy, perseverative or compulsive behaviour and hyperorality or dietary changes [1]. Patients presenting with at least three of these symptoms in the first 3 years of disease onset are diagnosed with the behavioural variant of frontotemporal dementia (bvFTD) [1]. Another clinical variant of FTD is the language variant, named primary progressive aphasia (PPA), and includes semantic variant (svPPA) and a non‐fluent variant (nfvPPA). Patients with FTD can also present a spectrum of other symptoms, including motor neuron disease (MND)/amyotrophic lateral sclerosis (ALS) [2]. Psychotic symptoms, such as delusions and hallucinations, are also commonly seen in bvFTD patients, during or prior to the onset of dementia [3]. The pathology of FTD, termed frontotemporal lobar degeneration (FTLD), is characterised by three subclasses of aggregated misfolded proteins: phosphorylated transactive response DNA binding protein 43 kDa (FTLD‐TDP), phosphorylated tau (FTLD‐tau) and fused in sarcoma (FTLD‐FUS) [4]. Several genetic autosomal dominant mutations are linked to FTD; a repeat expansion in the *C9orf72* gene and mutations in the progranulin (*GRN*) gene can lead to FTD with underlying TDP43 pathology, whereas mutations in the Microtubule Associated Protein Tau (*MAPT*) tau gene result in FTD with underlying tau pathology. Sporadic variants of TDP43 and tau are also prevalent, whereas patients with FTD due to FUS aggregation are described as only sporadic [4, 5, 6, 7, 8, 9].

We recently showed selective loss of the GABA receptor subunit theta (GABRQ) expressing cortical neurons in the anterior cingulate cortex (ACC) in bvFTD patients due to a *C9orf72* repeat expansion [10]. The GABRQ‐expressing neurons include the von Economo neurons (VENs), of which selective vulnerability has been linked to FTD in the earliest stages of the disease [11, 12, 13, 14, 15]. VENs have a unique morphology and are found mainly in Layer 5 of the human ACC and frontoinsular cortex (FI) [16]. They are distinguished from pyramidal neurons by their large bipolar cell body and thick dendrites [17]. VENs have been identified with a similar regional distribution in highly social mammals, such as primates, cetaceans and elephants, but are not found in common laboratory animals, such as mice and rats [18, 19, 20]. In addition, the GABRQ‐expressing population is also absent in mice [21]. Currently, it is not known whether the loss of GABRQ‐expressing neurons extends to other genetic and sporadic pathological forms of FTLD, including FTLD‐TDP, FTLD‐tau and FTLD‐FUS and how this relates to the early behavioural symptoms.

Here, we aim to investigate whether there is a loss of GABRQ‐expressing neurons in the ACC in the different pathological and genetic subtypes of FTD. In addition, we want to establish whether there is a link between the number of GABRQ‐expressing neurons and the early manifestation of behavioural symptoms.

## MATERIALS AND METHODS

### Subjects

Post‐mortem brain tissue was obtained from the Netherlands Brain Bank and the department of pathology, Amsterdam University Medical Centre (UMC), location VUmc, Amsterdam, Netherlands. During their disease course, donors were seen for diagnosis by neurologists experienced in neurodegenerative diseases at either the Alzheimer Centre, Amsterdam UMC, location VUmc, or the Erasmus Medical Centre, Rotterdam, Netherlands. We included FTLD donors based on their main pathological diagnosis with the three main pathological subtypes: 34 FTLD‐TDP (including 6 with a *GRN* mutation [TDP‐GRN], 16 with a *C9orf72* repeat expansion [TDP‐C9] and 12 with sporadic pathology [TDP‐SP]), 14 FTLD‐tau donors (10 with a *MAPT P301L* mutation [tau‐MAPT] and 4 with sporadic tau pathology [tau‐SP]) and 8 FTLD‐FUS donors. We compared these to donors with typical amnestic presentation of Alzheimer's disease (AD) (*n* = 7) and age‐matched neurologically unaffected controls (*n* = 12) (Tables 1 and S1). Donors with extensive concomitant pathology (non‐age related AD pathology or extensive pathology throughout the cortex that was not related to their main pathological diagnosis) were excluded from the study.

**TABLE 1:** Demographic and clinical data of donors included in the study

	*n*	Gender m/f	Age at death (years)	Disease duration (years)
Control	12	6/6	66.9 (51–86)	N/A
FTLD‐TDP	34	13/21	64.7 (40–81)	6.9 (2–18)
TDP‐SP	12	6/6	66.8 (50–81)	8 (2–16)
TDP‐C9	16	6/10	64.2 (40–77)	6.9 (2–18)
TDP‐GRN	6	1/5	61.7 (51–76)	4.7 (3–7)
FTLD‐tau	14	8/6	62.6 (46–82)	9.4 (2–23)
tau‐SP	4	2/2	71.3 (65–82)	8.5 (4–15)
tau‐MAPT	10	6/4	59.2 (46–66)	9.8 (2–23)
FTLD‐FUS	8	6/2	54.8 (41–68)	8.7 (2–19)
AD	7	3/4	82.3 (69–98)	9.3 (4–15)
*P*		0.57^a^	0.01^b^	0.50^b^

*Note*: Values are an average mean (with range). *P* values are calculated based on group comparisons between control, FTLD‐TDP, FTLD‐tau, FTLD‐FUS and AD.

Abbreviations: AD, Alzheimer's disease; C9, *C9orf72*; f, female; FTD, frontotemporal dementia; GRN, progranulin; m, male; MAPT, Microtubule Associated Protein Tau; N/A, not applicable; SP, sporadic.

^a^ Kruskal‐Wallis.

^b^ ANOVA.

### Immunohistochemical procedures and GABRQ quantification

Samples from the ACC were collected and processed using immunohistochemistry as described previously [10]. Briefly, the right hemisphere was fixed for 4 weeks in PFA. The ACC was dissected perpendicular to the corpus callosum, caudal to the genu and embedded in paraffin. Ten micrometre‐thick sequential sections were cut, and immunohistochemical analysis was performed with GABRQ (1:750, HPA002063; Sigma Aldrich) and GABA receptor subunit epsilon (GABRE 1:1,000: HPA045918, Sigma Aldrich, St. Louis, MO) to determine Layer 5 borders. To quantify GABRQ expression, we analysed the GABRQ stained slides of the ACC using Stereoinvestigator software (V11.6.2). Within Layer 5, all neurons were counted using the Meander scan option in Stereoinvestigator and divided into four categories: (1) GABRQ‐expressing VENs, (2) GABRQ‐expressing pyramidal neurons, (3) GABRQ‐negative VENs and (4) GABRQ‐negative pyramidal neurons. VENs were identified based on their typical morphological profile [22]: a long elongated soma with one apical and one basal dendrite. In contrast, pyramidal neurons were identified based on their more rounded or tear drop soma with two basal dendrites and were counted if their cell body was larger than 10 μm. As the slides were cut perpendicular to the pia and the GABRQ‐immunoreactivity revealed the clear morphology of neurons, the classification into neuronal groups was successful for most neurons. However, some neurons were encountered where classification was challenging and are listed separately (Table S2).

### Clinical information

For all donors, extensive clinical information was available. The neurological symptoms were evaluated by a neurologist (M.S.). Donors presented varying phenotypes, including mild behavioural symptoms and prominent language symptoms, to more prominent behavioural symptoms (see Table S1 for clinical diagnosis). The behavioural symptoms were scored using the clinical criteria of Rascovsky et al. [1]. The framework consists of six main categories each with two or three subcategories. If a behavioural symptom was present in the first 3 years of disease onset, it was scored as present (1) or absent (0). If symptoms were not explicitly mentioned and could not be extrapolated from the records, they were considered absent. The sum of the behavioural scores resulted in a behavioural score ranging from 0–14.

### Statistics

Statistics were performed using SPSS 22. Pearson correlation was used to assess relation between clinical profile and scores for neurodegeneration, and differences between groups were assessed using one‐way ANOVA with Tukey's post hoc analysis.

## RESULTS

### FTLD‐TDP and FTLD‐FUS but not FTLD‐tau donors show loss of GABRQ‐expressing neurons

**FIGURE 1:**
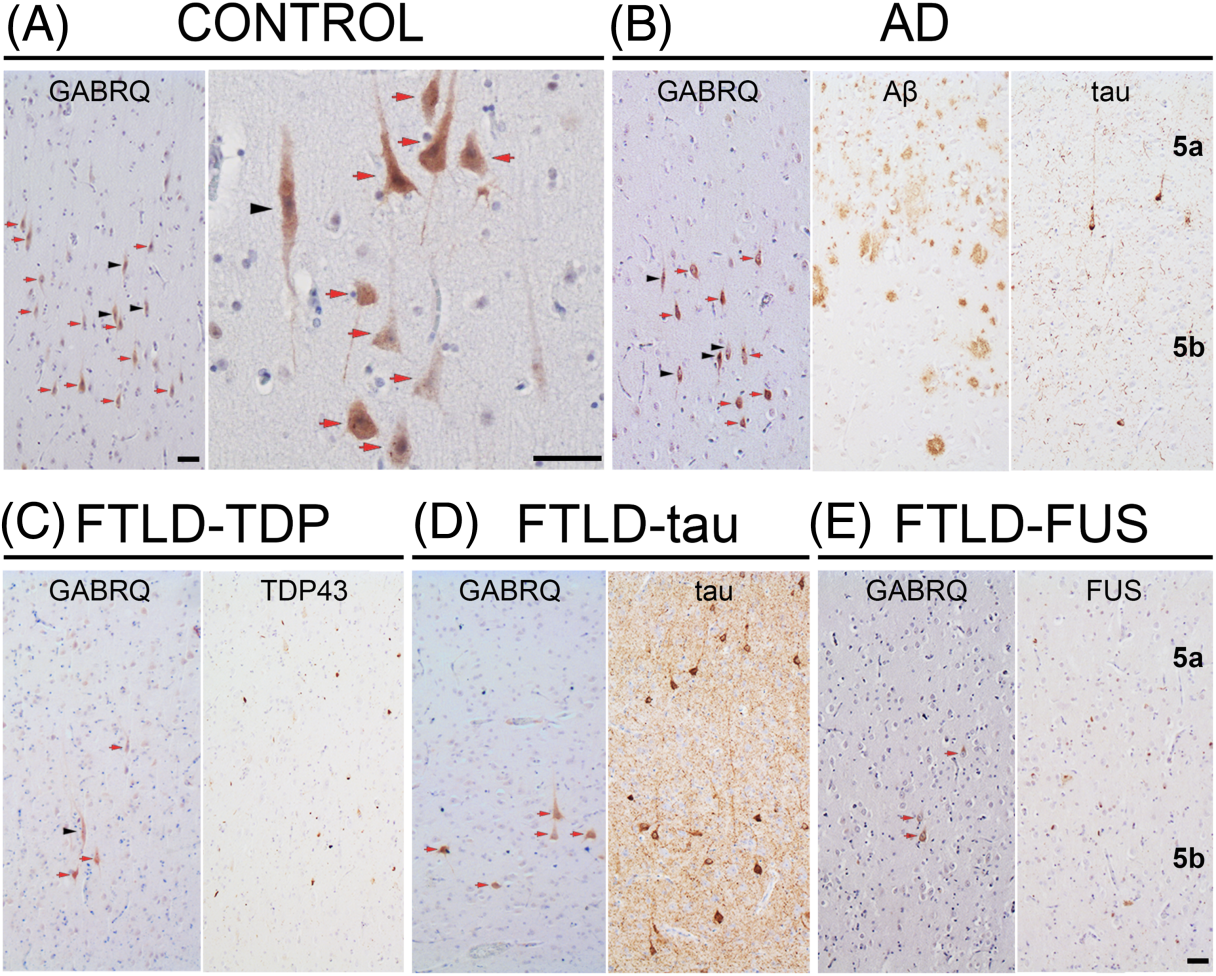
GABRQ immunopositive neurons in the ACC. GABRQ immunostaining is seen in VENs (black arrowhead) and surrounding pyramidal neurons (red arrow). Layer 5 GABRQ‐immunopositive neurons are seen in controls (A), whereas a reduced number of GABRQ‐positive neurons can be seen in donors with underlying TDP43 (C), tau (D) and FUS (E) pathology. In AD, a similar expression pattern of GABRQ‐positive neurons can be seen when compared to control (B). Adjacent images show pathology for each disease group. The same cases were used to denote typical GABRQ expression and pathology seen in each disease group (A, control case 11; B, AD case 3; C, FTLD‐TDP‐GRN case 6; D, FTLD‐tau‐MAPT case 9; E, FTLD‐FUS case 1). Scale bars represent 50 μm

**FIGURE 2:**
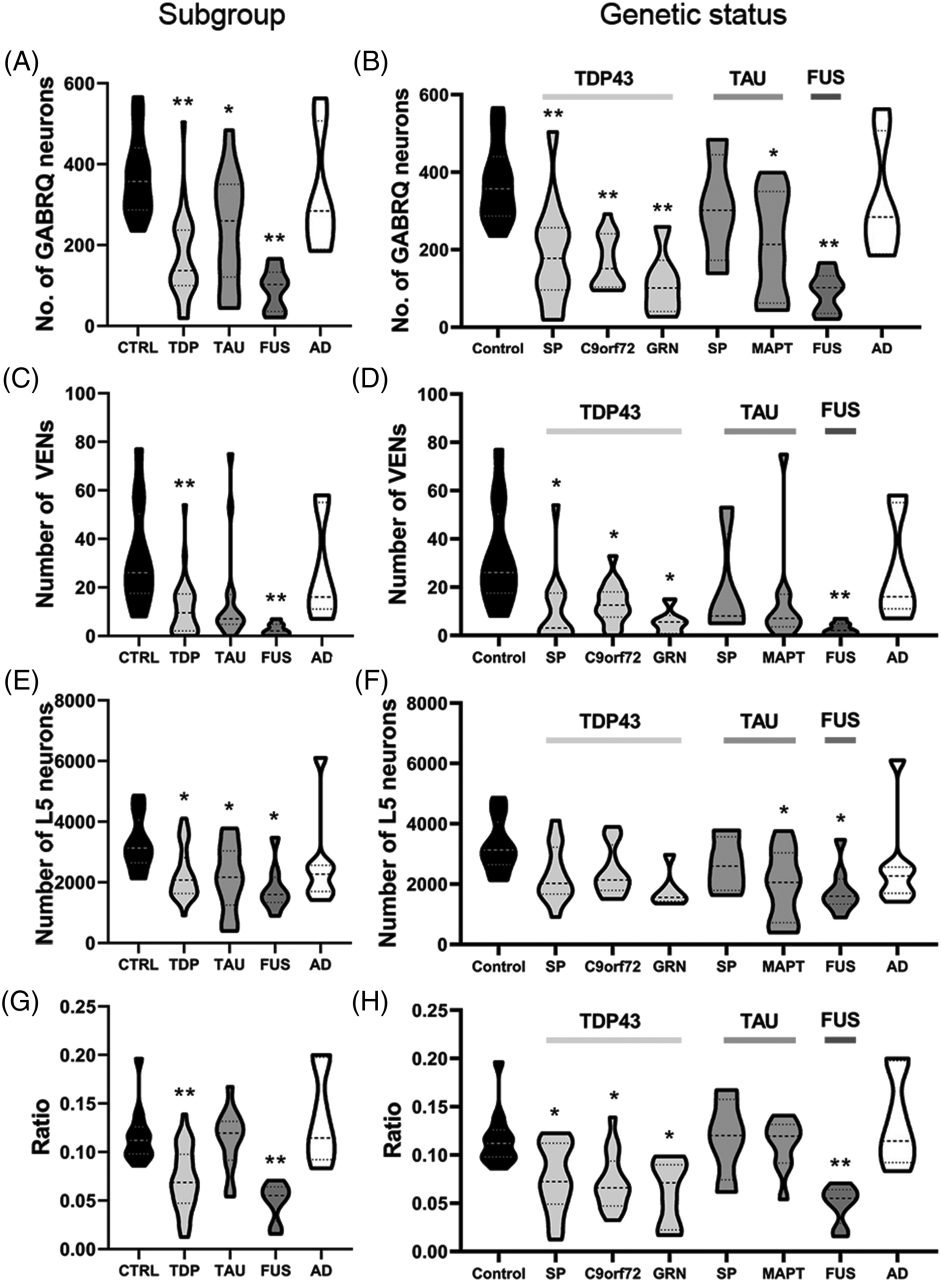
The number of VENs and GABRQ‐expressing pyramidal neurons and ratio of the GABRQ‐expressing population over the total Layer 5 neuronal population. Number of neurons and GABRQ/total L5‐ratio per molecular subgroup (A, C, E, G) and genetic status (B, D, F, H). All statistical analysis was performed against control group. The number of GABRQ‐expressing pyramidal neurons is reduced in all three FTD subtypes (A); however, when split based on genetic status sporadic tau, donors do not show a significant decrease (B). There is a reduction in number of VENs in all FTD subtypes, with donors with TDP43 and FUS pathology reaching significance (C, D). A significant reduction in total Layer 5 neurons is seen in all FTD donors (E); however, when split genetically, only those with a *MAPT* mutation and FUS reach significance (F). A significant reduction in GABRQ/total L5‐ratio of GABRQ‐expressing neurons is seen in all FTD donors with TDP43 pathology and FTLD‐FUS donors when compared to control (G, H). In all comparisons, the number of neurons and GABRQ/total L5‐ratio in AD donors remain similar to control. Shown are mean levels ±SD. ^*^ *p <* 0.05; ^**^ *p* < 0.01

We quantified the number of GABRQ‐expressing pyramidal neurons in the ACC (Figure 1). See Table S2 for the raw data. A significant difference in GABRQ‐expressing pyramidal neurons was seen between all groups (*F*[4] = 11.49, *p* < 0.001). Post hoc testing revealed that compared to controls, there was a difference in FTLD‐TDP (*p* < 0.001), FTLD‐tau (*p* = 0.01) and FTLD‐FUS (*p* < 0.001) subgroups, but not in AD (*p* = 0.92) (Figure 2A). When we investigated in greater detail the number of GABRQ‐expressing neurons per genetic status, the number of GABRQ‐expressing neurons in donors with underlying TDP43 pathology, including those with a *C9orf72* repeat expansion, or *GRN* mutation, were all significantly lower compared to controls (all: *p* < 0.001). In donors with underlying tau pathology, the sporadic donors did not show a significant decrease (*p* = 0.98) compared to control, whereas donors with *MAPT* mutation did (*p* = 0.04). No difference was seen in AD donors (*p* = 0.99) (Figure 2B). This indicates that all FTD donors, except sporadic FTLD‐tau donors, show a reduction in GABRQ‐expressing pyramidal neurons, whereas this cell population is spared from neurodegeneration in AD.

Overall, the number of VENs and GABRQ‐expressing pyramidal neurons showed a significant correlation across all donors included in this study (*n* = 75: *p* = 0.69, *p* < 0.001). The number of VENs shows a similar decrease as GABRQ‐expressing neurons. When comparing the percentage of GABRQ‐immunopositive VENs over total VENs across groups, no significant difference is observed (*F*[4] = 2.08, *p* = 0.09), suggesting there is a similar proportion of GABRQ‐negative VENs in all groups. A significant difference was seen in the number of VENs between molecular groups (*F*[4] = 6.43, *p* < 0.001), and compared to controls, the FTLD‐TDP and FTLD‐FUS groups showed a significantly lower number of VENs (*p* = 0.001 for both), which was not seen in FTLD‐tau (*p* = 0.06) and AD (*p* = 0.96) (Figure 2C). In the FTLD‐TDP group, the number of VENs was significantly lower in sporadic (*p* = 0.03), *C9orf72* (*p* = 0.04) and *GRN* (*p* = 0.03) donors compared to controls. In donors with tau pathology, the sporadic donors (*p* = 0.79) and *MAPT P301L* carriers (*p* = 0.15) showed no difference compared to control (Figure 2D).

A significant reduction in the total Layer 5 neuronal population was seen in all FTD donors compared to controls (*F*[4] = 3.53, *p* = 0.01; FTLD‐TDP: *p* = 0.03, FTLD‐tau: *p* = 0.04, FTLD‐FUS: *p* = 0.01 and AD: *p* = 0.66); however, when grouped based on genetic status, only tau‐MAPT donors and those with FUS pathology reached significance (*p* = 0.05 and *p* = 0.01, respectively) (Figure 2E,F).

The ratio of GABRQ‐expressing neurons was calculated by dividing the total number of GABRQ‐expressing neurons over the total Layer 5 neuronal population (GABRQ/total L5‐ratio). This reflects the specific loss of GABRQ‐immunoreactive neurons, as all groups show neuronal loss in an advanced disease state. Using an ANOVA, a significant difference was found in GABRQ/total L5‐ratio between the groups (*F*[4] = 12.82, *p* < 0.001). Tukey post hoc analysis revealed a significant decline of GABRQ/total L5‐ratio in donors with FTLD‐TDP (*p* = 0.001) and FTLD‐FUS (*p* < 0.001) compared to controls, but not in FTLD‐tau (*p* = 0.99) and AD (*p* = 0.78) (Figure 2G). When looking at the genetic status, all TDP groups reached statistical significance (TDP‐SP: *p* = 0.046, TDP‐C9: *p* = 0.011; TDP‐GRN: *p* = 0.019). In contrast, both FTLD‐tau groups were not significantly different in GABRQ/total L5‐ratio compared to control (*p* = 1.000 for both) (Figure 2H). When we look at the GABRQ/total L5‐ratio between pathological subtypes within the FTLD‐TDP group, no difference was found between the subtypes A, B, C and E (*F*[3] = 0.680, *p* = 0.57).

### Relation between VENs and GABRQ‐expressing neurons and behavioural symptoms

**FIGURE 3:**
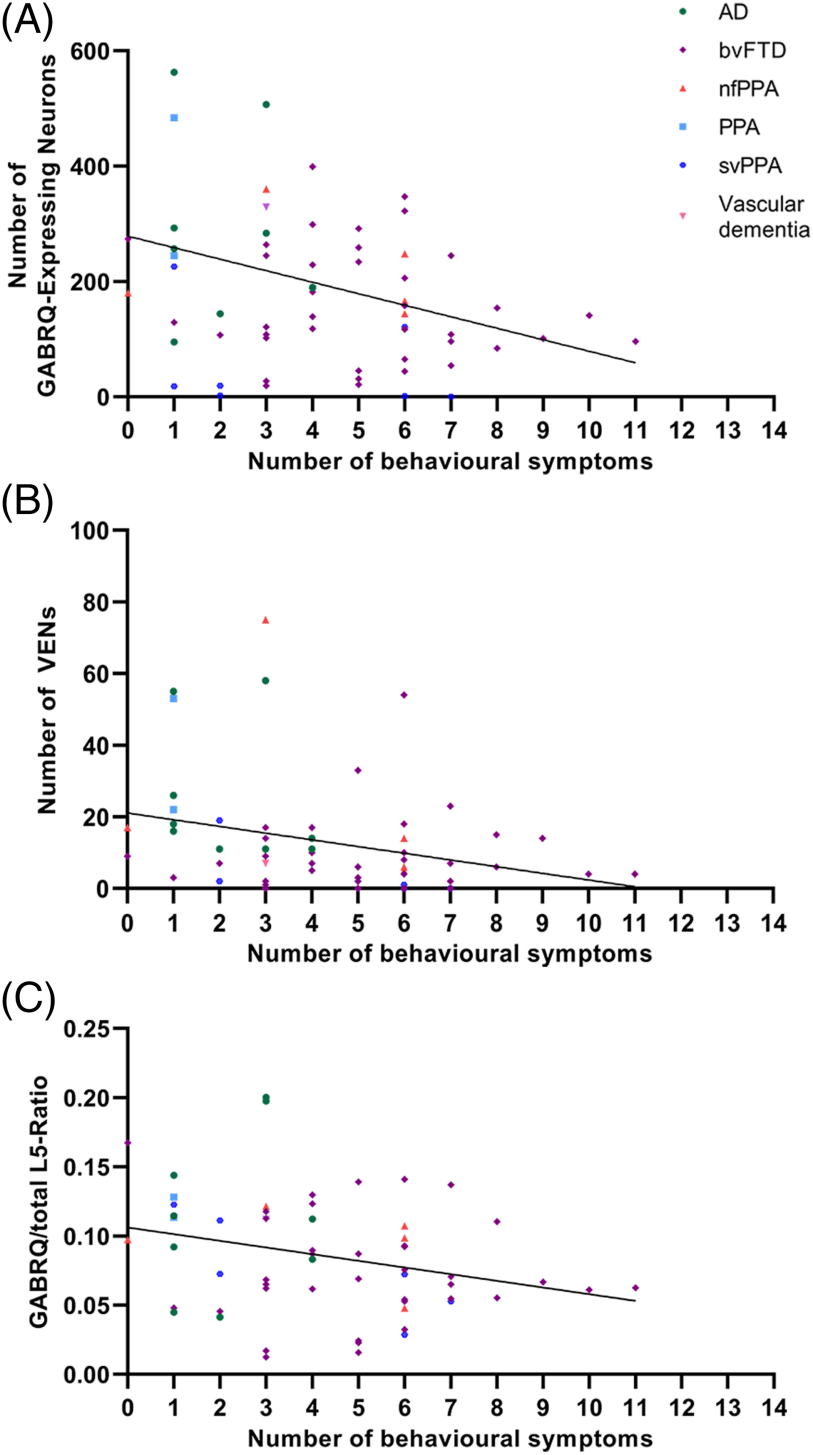
The correlation of behavioural symptoms present in the first 3 years of disease onset with the number of GABRQ‐expressing neurons, VENs and GABRQ/total L5‐ratio in all FTLD and AD donors (*n* = 63). A negative correlation is seen across all FTLD groups. The greater the number of behavioural symptoms seen in donors was associated with a lower number of GABRQ‐expressing neurons (*r* = −0.29, *p* < 0.001, A) and VENs (*r* = −0.37, *p* = 0.003, B). As expected, a lower GABRQ/total L5‐ratio also correlated with a higher score of behavioural symptoms (*r* = −0.27, *p* = 0.03, C)

We investigated the presence of behavioural symptoms in the first 3 years of disease onset in FTD and AD donors in relation to the GABRQ‐expressing neurons. The average disease duration of all FTD donors was 7.83 years. The first 3 years were used as the time point since the clinical diagnoses of bvFTD are made on the presence of behavioural symptoms seen in this time period, and the symptoms are therefore well recorded. The control donors had no mention of behavioural problems. The presence of behavioural symptoms per donor was accumulated over the categories to reflect increased behavioural symptomology. We found that a lower number of GABRQ‐expressing neurons correlated with a higher score on early behavioural symptoms (*n* = 63: *r* = −0.39, *p* < 0.001) (Figure 3A). As expected, the number of VENs and GABRQ/total L5‐ratio showed similar correlation (*r* = −0.37, *p* = 0.003 and *r* = −0.27, *p* = 0.035, respectively) (Figure 3B,C).

## DISCUSSION

Here, we show a loss of VENs and the GABRQ‐expressing neuronal population in FTD donors with underlying TDP43 and FUS pathology, but not in our donors with tau pathology. The number of VENs and GABRQ‐expressing neurons is significantly lower in FTD groups, but not in AD, indicating that these neurons are specifically linked with FTD. When looking at the clinical presentation of FTD, donors with more behavioural symptoms during the first 3 years of their disease course show a lower GABRQ/total L5‐ratio, supporting the hypothesis that this neuronal population is related to the behavioural presentation in FTD.

### A loss of GABRQ‐expressing neurons is observed in donors with FTLD‐TDP and FTLD‐FUS molecular subclasses

In our analysis, we have shown that all FTD donors, except sporadic FTLD‐tau, have a lower number of VENs and GABRQ‐expressing neurons in the ACC compared to control and AD donors. In donors with underlying TDP43 and FUS pathology, a lower ratio of GABRQ‐expressing neurons over the total Layer 5 neuronal population is found, but not in the FTLD‐tau group. Possible explanations for this finding could be of a clinical nature, as behavioural symptoms are not as prominent in FTLD‐tau donors [23], or due to the low number of sporadic FTLD‐tau cases available for analysis. In addition, VENs display early pathology in FTLD‐TDP donors, and in donors with *MAPT* mutations *V337 M*, *IVS10 + 16* and *A152T*, although not in a donor with the *P301L* mutation [24, 25], suggesting that not all genetic forms of FTLD‐*MAPT* show similar patterns of selective vulnerability. As our genetic FTLD‐tau group only consisted of *P301L* carriers, this could explain why we do not see loss of VENs and GABRQ‐expressing neurons. Future studies should explore different *MAPT* mutations and a larger number of sporadic donors with prominent behavioural changes. The number of GABRQ‐expressing neurons and VENs in FTLD‐tau was relatively high, whereas neuronal Layer 5 number was similar compared to the other FTLD groups, suggesting there is neuronal loss without specifically targeting the GABRQ‐expressing neuronal population. Interestingly, in AD, the GABRQ‐expressing neuronal population is selectively spared, showing a similar GABRQ/total L5‐ratio compared to controls. This is similar to what has already been reported in VENs [10, 11, 12, 13, 14]. Overall, TDP43 and FUS pathology show a selective loss of the GABRQ‐expressing neuronal population in the ACC in contrast to our donors with tau pathology where a more global atrophy is observed. TDP43 and FUS are ubiquitously expressed heterogeneous nuclear riboproteins (hnRNPs) that regulate RNA splicing and are also involved in mRNA transport, stability and RNA translation [26, 27, 28, 29]. In FTLD‐TDP and FTLD‐FUS, TDP43 and FUS, respectively, are depleted from the nucleus and aggregate in the cytoplasm. Sequestration of these proteins from the nucleus has profound effects on the cell that could ultimately result in neuronal death [30, 31]. Based on our data, it is likely that the GABRQ‐expressing neuron and VEN loss are a consequence of upstream modulators of hnRNPs.

### Early behavioural symptoms correlate with the number of GABRQ‐expressing neurons

We found that the number of VENs and GABRQ‐expressing neurons present at the end‐stage of the disease correlate with an early onset of complex behavioural profiles. In our cohort, there are donors with bvFTD but also those that present with the language and movement variants of FTD. Donors with less prominent behavioural changes show a higher number of VENs and GABRQ‐expressing neurons. It cannot be overlooked that at the time of death most donors are at the end stage of their disease showing increasing number and intensity of symptoms. Since the ACC is affected early in disease and continues to degenerate throughout a donor's disease course [32], we can assume that the loss of the GABRQ‐expressing neuronal population in the ACC occurs proportionally to the disease duration and therefore focussing on the initial stages can provide insight into the anatomical basis of symptoms. In addition, previous work has shown that the FI also shows selective loss of VENs and fork cells in the early stages FTD [14], and for future research, it would be interesting to explore the GABRQ‐neuronal population in this region.

One important consideration is that this study is limited to the expression of one marker, GABRQ. In our data, we see that the percentage of GABRQ‐negative VENs over total VENs increases in FTLD. This could indicate that either the GABRQ‐negative VENs are less likely to degenerate or that some GABRQ‐expression is lost in FTLD without neurodegeneration. If the latter is the case, this would also reflect on the pyramidal population, leading to an overestimation of neuronal loss in FTLD. However, the possibility remains that the neurons that lost expression of GABRQ will also have altered functioning. Future functional studies should investigate this in more detail.

To conclude, TDP43 and FUS pathology show a reduction of the GABRQ‐expressing neuronal population in the ACC. Donors from our FTLD‐tau group (sporadic and *MAPT P301L* mutation) showed no selective reduction of the GABRQ‐expressing neuronal population, but this has to be explored across more pathological and clinical variants of FTLD‐tau. The severity of behavioural symptoms is correlated with the GABRQ‐expressing neuronal population, suggesting that this neuronal population is a key modulator of behaviour in FTD.

## CONFLICT OF INTEREST

The authors declare no conflict of interest.

## AUTHOR CONTRIBUTIONS

AAD and PGP designed the study and wrote the manuscript. PGP, MS and AAD performed the experiments and quantification of the data. PGP and AAD analysed the data. JCvS, ABS, YALP and JJHM provided intellectual contribution and participated in discussion. FHB, BDCB, AJMR and NBB were responsible for the autopsy, storage of the post‐mortem material and clinical and neuropathological evaluation. NBB also assisted in the selection of suitable tissue from its bank. All authors read and approved the final manuscript.

## ETHICS STATEMENT

All procedures performed in the study were in accordance with the ethical standards of Amsterdam University Medical Centre location VUmc. Ethical approval for the NBB procedures and forms was given by the Medical Ethics Committee of the VU University Medical Centre (Amsterdam, Netherlands). Informed consent and tissue collection were also carried out in accordance with the Code of conduct for Brain Banking and Declaration of Helsinki [33].

## INFORMED CONSENT

All donors gave informed consent for autopsy, storage and use of their tissue and anonymised clinical and neuropathological data for research purposes.

### PEER REVIEW

The peer review history for this article is available at https://publons.com/publon/10.1111/nan.12798.

## Supporting information


**Table S1.** Supporting Information

**Table S2.** Supporting Information

## Data Availability

The data that support the findings of this study are available from the corresponding author upon reasonable request.
