# Supplementary material for: The severity of behavioural symptoms in FTD is linked to the loss of GABRQ‐expressing VENs and pyramidal neurons
Source: Neuropathol Appl Neurobiol. 2022 Feb 24;48(4):e12798. doi: 10.1111/nan.12798 (PMC9306948; doi:10.1111/nan.12798)
Supplement: Supplementary file 1 — Table S1. Supporting Information [file NAN-48-0-s001.docx]

|  | **Age of symptom onset** | **Age at Death** | **Gender**  **(M / F)** | **Brain weight (gram)** | **PMD (Hrs:min)** | **Path. subtype** | **ABC score** | **Copath.** | **Clinical Diagnosis** |
| --- | --- | --- | --- | --- | --- | --- | --- | --- | --- |
| 1. Control | - | 64 | F | 1159 | 08:35 |  | 2,0,0 |  | - |
| 1. Control | - | 55 | M | 1393 | 07:15 |  | 2,0,0 |  | - |
| 1. Control | - | 64 | F | 1221 | 05:40 |  | 0,1,0 |  | - |
| 1. Control | - | 60 | F | 1310 | 08:10 |  | 0,0,0 |  | - |
| 1. Control | - | 83 | M | 1108 | 05:05 |  | 2,1,0 |  | - |
| 1. Control | - | 60 | F | 1215 | 05:30 |  | 0,0,0 |  | - |
| 1. Control | - | 76 | F | 1047 | 05:30 |  | 1,1,0 |  | - |
| 1. Control | - | 68 | M | 1223 | <12:00 |  | 0,0,0 |  | - |
| 1. Control | - | 70 | F | 1235 | <12:00 |  | 2,1,0 |  | - |
| 1. Control | - | 86 | M | 1360 | <12:00 |  | 0,1,0 |  | - |
| 1. Control | - | 51 | M | 1470 | <12:00 |  | 2,1,0 |  | - |
| 1. Control | - | 53 | M | 1257 | <12:00 |  | 1,0,0 |  | - |
|  |  |  |  |  |  |  |  |  |  |
| **FTLD-TDP** |  |  |  |  |  |  |  |  |  |
| 1. TDP-SP | 53 | 63 | M | 1190 | 10:30 | TDP-C | 0,0,0 |  | svPPA |
| 1. TDP-SP | 52 | 64 | F | 975 | 06:25 | TDP-C | 1,0,0 |  | bvFTD |
| 1. TDP-SP | 66 | 74 | F | 1044 | 04:00 | TDP-C | 0,1,0 |  | svPPA |
| 1. TDP-SP | 56 | 68 | M | 1162 | 07:00 | TDP-C | 2,1,0 |  | svPPA |
| 1. TDP-SP | 62 | 64 | M | 1071 | 04:29 | TDP-E | 2,1,0 |  | svPPA |
| 1. TDP-SP | 69 | 72 | F | 1135 | 04:10 | TDP-C | 1,1,0 |  | svPPA |
| 1. TDP-SP | 58 | 60 | M | 1159 | 05:00 | TDP-E | 0,0,0 |  | bvFTD |
| 1. TDP-SP | 65 | 69 | M | 1075 | 09:00 | TDP-E | 2,0,0 |  | svPPA |
| 1. TDP-SP | 50 | 50 | F | 1052 | 05:19 | TDP-A | 1,1,0 |  | svPPA |
| 1. TDP-SP | 61 | 76 | M | 955 | 06:55 | TDP-A | 1,1,0 |  | bvFTD |
| 1. TDP-SP | 75 | 81 | F | 955 | 04:10 | TDP-A | 1,1,0 |  | bvFTD with parkinsonism |
| 1. TDP-SP | 55 | 61 | F | 755 | 06:30 | TDP-A | 0,0,0 |  | bvFTD |
|  |  |  |  |  |  |  |  |  |  |
| 1. TDP-C9 | 67 | 72 | F | 955 | 04:50 | TDP-B | 1,1,0 |  | bvFTD |
| 1. TDP-C9 | 38 | 42 | F | 1061 | 05:45 | TDP-B | 0,0,0 |  | FTD-ALS (bvFTD) |
| 1. TDP-C9 | 69 | 75 | M | 1086 | 06:25 | TDP-A | 0,1,0 |  | bvFTD |
| 1. TDP-C9 | 61 | 68 | F | 825 | 06:20 | TDP-B | 0,2,0 |  | PPA |
| 1. TDP-C9 | 35 | 40 | F | 840 | 05:50 | TDP-B | 0,1,0 |  | CBS (bvFTD) |
| 1. TDP-C9 | 54 | 60 | M | 1065 | 05:05 | TDP-B | 0,1,0 |  | bvFTD |
| 1. TDP-C9 | 50 | 70 | F | 958 | 04:40 | TDP-A | 2,2,1 |  | bvFTD |
| 1. TDP-C9 | 59 | 67 | F | 886 | 06:45 | TDP-B | 2,2,2 |  | AD |
| 1. TDP-C9 | 46 | 64 | M | 1075 | 04:35 | TDP-B | 1,1,0 |  | bvFTD |
| 1. TDP-C9 | 73 | 75 | M | 1480 | 17:35 | TDP-B | 2,2,1 |  | bvFTD |
| 1. TDP-C9 | 45 | 52 | F | 719 | 08:16 | TDP-B | 0,1,0 |  | bvFTD |
| 1. TDP-C9 | 62 | 66 | F | 1099 | 05:25 | TDP-B | 2,2,1 |  | nfPPA |
| 1. TDP-C9 | 70 | 77 | F | 851 | 04:25 | TDP-A | 1,1,0 |  | AD |
| 1. TDP-C9 | 45 | 59 | M | 1170 | 08:00 | TDP-B | 0,1,0 |  | bvFTD |
| 1. TDP-C9 | 69 | 75 | F | 924 | 05:15 | TDP-A | 0,1,0 |  | bvFTD |
| 1. TDP-C9 | 63 | 65 | M | 1120 | 05:45 | TDP-E | 1,1,0 |  | nfPPA |
|  |  |  |  |  |  |  |  |  |  |
| 1. TDP-GRN | 60 | 66 | F | 894 | 05:15 | TDP-A | 0,1,0 |  | nfPPA |
| 1. TDP-GRN | 47 | 51 | F | - | - | TDP-A | 0,1,0 |  | bvFTD |
| 1. TDP-GRN | 73 | 76 | F | 1076 | 03:35 | TDP-A | 2,2,1 |  | bvFTD |
| 1. TDP-GRN | 45 | 52 | F | 960 | 03:50 | TDP-A | 0,1,0 |  | bvFTD |
| 1. TDP-GRN | 54 | 58 | M | 917 | 06:00 | TDP-A | 0,0,0 |  | bvFTD |
| 1. TDP-GRN | 63 | 67 | F | 1045 | 04:15 | TDP-A | 0,1,0 |  | bvFTD |
|  |  |  |  |  |  |  |  |  |  |
| **FTLD-Tau** |  |  |  |  |  |  |  |  |  |
| 1. Tau-SP | 58 | 65 | F | 1130 | 04:25 | undefined* | 0,2,0 |  | bvFTD |
| 1. Tau-SP | 68 | 72 | M | 1341 | 08:14 | undefined* | 1,1,0 |  | bvFTD |
| 1. Tau-SP | 74 | 82 | M | 1012 | 04:10 | Pick’s | 2,2,0 |  | Vascular dementia |
| 1. Tau-SP | 51 | 66 | F | 1008 | 05:25 | CBD | 1,0,0 |  | PPA |
|  |  |  |  |  |  |  |  |  |  |
| - - - 1. Tau-MAPT | 53 | 64 | M | 1029 | 06:25 |  | 0,1,0 |  | bvFTD |
| - - - 1. Tau-MAPT | 58 | 66 | F | 752 | 06:40 |  | 0,1,0 |  | bvFTD |
| - - - 1. Tau-MAPT | 49 | 52 | M | 1115 | 11:30 |  | 0,1,0 |  | bvFTD |
| - - - 1. Tau-MAPT | 51 | 60 | M | 977 | 05:23 |  | 0,0,0 |  | nfPPA |
| - - - 1. Tau-MAPT | 36 | 59 | F | 1079 | 05:50 |  | 1,1,0 |  | bvFTD |
| - - - 1. Tau-MAPT | 58 | 66 | F | 792 | 05:00 |  | 0,1,0 |  | bvFTD |
| - - - 1. Tau-MAPT | 39 | 46 | M | 1220 | 05:35 |  | 0,1,0 |  | bvFTD |
| - - - 1. Tau-MAPT | 45 | 64 | F | 652 | 07:30 |  | 0,1,0 |  | bvFTD |
| - - - 1. Tau-MAPT | 53 | 55 | M | 1100 | 04:25 |  | 0,0,0 |  | bvFTD |
| - - - 1. Tau-MAPT | 52 | 60 | M | 1300 | 05:50 |  | 1,0,0 |  | bvFTD |
|  |  |  |  |  |  |  |  |  |  |
| **FTLD-FUS** |  |  |  |  |  |  |  |  |  |
| 1. FUS | 30 | 41 | F | 1040 | 05:10 | BIBD | 0,0,0 |  | bvFTD |
| 1. FUS | 49 | 68 | F | 1224 | 04:15 | aFTLD-U | 1,1,0 |  | bvFTD |
| 1. FUS | 41 | 47 | M | 1328 | 05:25 | aFTLD-U | 0,0,0 |  | bvFTD |
| 1. FUS | 44 | 50 | M | 1010 | 06:55 | aFTLD-U | 0,0,0 |  | bvFTD |
| 1. FUS | 50 | 52 | M | 1240 | 03:35 | aFTLD-U | 0,1,0 |  | nfPPA |
| 1. FUS | 73 | 76 | M | 1205 | 07:30 | aFTLD-U | 0,0,0 |  | bvFTD |
| 1. FUS | 40 | 54 | M | 1065 | 06:05 | NIFID | 0,2,0 |  | bvFTD |
| 1. FUS | 52 | 59 | M | 1010 | 03:40 | aFTLD-U | 0,1,0 |  | bvFTD |
|  |  |  |  |  |  |  |  |  |  |
| **AD** |  |  |  |  |  |  |  |  |  |
| - - - 1. AD | 66 | 78 | F | 1130 | 04:55 |  | 3,3,3, |  | AD |
| - - - 1. AD | 83 | 87 | M | 1385 | 08:45 |  | 3,3,3 | TDP limbic | AD |
| - - - 1. AD | 90 | 98 | M | 1095 | 04:25 |  | 3,3,3 | LB Amygdala | AD |
| - - - 1. AD | 60 | 69 | F | 1065 | 06:10 |  | 3,3,3 |  | AD |
| - - - 1. AD | 80 | 89 | F | 940 | 05:45 |  | 3,3,3 | LB -brainstem  TDP Limbic | AD |
| - - - 1. AD | 67 | 82 | M | 1205 | 07:30 |  | 3,3,3 | LB Amygdala  TDP Limbic | AD |
| - - - 1. AD | 63 | 73 | F | 1290 | 07:00 |  | 3,3,3 |  | AD |
|  |  |  |  |  |  |  |  |  |  |
| FTLD–frontotemporal lobe dementia; TDP-TAR DNA-binding protein 43; SP–sporadic; C9-C9orf72; GRN–progranulin; MAPT-Microtubule Associated Protein Tau; AD-Alzheimer’s disease; m-male; f-female; svPPA-semantic variant primary progressive aphasia; nfvPPA-non-fluent variant primary progressive aphasia; bvFTD-behavioural variant frontotemporal dementia; CBS-corticobasal syndrome; ALS-Amyotrophic lateral sclerosis; - -not applicable/not available/not scored; *undefined, possible de novo; LB-Lewy bodies | | | | | | | | | |
