# Supplementary material for: The severity of behavioural symptoms in FTD is linked to the loss of GABRQ‐expressing VENs and pyramidal neurons
Source: Neuropathol Appl Neurobiol. 2022 Feb 24;48(4):e12798. doi: 10.1111/nan.12798 (PMC9306948; doi:10.1111/nan.12798)
Supplement: Supplementary file 2 — Table S2. Supporting Information [file NAN-48-0-s002.docx]

| **Case no. Neuropathological diagnosis** | **GABRQ Positive VENs** | **GABRQ Positive Pyr** | **GABRQ Negative VENs** | **GABRQ Negative Pyr** | **Unsure** | **All Neurons** | **GABRQ positive VENs & Pyr / total L5 neuron ratio** |
| --- | --- | --- | --- | --- | --- | --- | --- |
|  |  |  |  |  |  |  |  |
| 1. Control | **72** | **381** | 5 | 4126 | 0 | 4584 | **0.10** |
| 1. Control | **24** | **344** | 4 | 2763 | 5 | 3140 | **0.12** |
| 1. Control | **8** | **309** | 0 | 2369 | 0 | 2686 | **0.12** |
| 1. Control | **43** | **359** | 12 | 2710 | 0 | 3124 | **0.13** |
| 1. Control | **22** | **213** | 2 | 1947 | 0 | 2184 | **0.11** |
| 1. Control | **17** | **266** | 0 | 2604 | 0 | 2887 | **0.10** |
| 1. Control | **24** | **492** | 0 | 2111 | 0 | 2627 | **0.20** |
| 1. Control | **28** | **246** | 4 | 2913 | 0 | 3191 | **0.09** |
| 1. Control | **35** | **263** | 1 | 1832 | 0 | 2131 | **0.14** |
| 1. Control | **12** | **384** | 0 | 3840 | 0 | 4236 | **0.09** |
| 1. Control | **19** | **547** | 0 | 4301 | 0 | 4867 | **0.12** |
| 1. Control | **57** | **289** | 2 | 3175 | 0 | 3523 | **0.10** |
|  |  |  |  |  |  |  |  |
| **FTLD-TDP** |  |  |  |  |  |  |  |
| 1. TDP-SP | **0** | **88** | 0 | 1581 | 0 | 1669 | **0.05** |
| 1. TDP-SP | **2** | **232** | 1 | 3162 | 0 | 3397 | **0.07** |
| 1. TDP-SP | **1** | **25** | 0 | 886 | 0 | 912 | **0.03** |
| 1. TDP-SP | **1** | **126** | 1 | 1626 | 0 | 1754 | **0.07** |
| 1. TDP-SP | **0** | **121** | 0 | 1551 | 0 | 1672 | **0.07** |
| 1. TDP-SP | **18** | **486** | 0 | 3605 | 0 | 4109 | **0.12** |
| 1. TDP-SP | **54** | **268** | 0 | 3145 | 0 | 3467 | **0.09** |
| 1. TDP-SP | **17** | **213** | 2 | 1836 | 0 | 2068 | **0.11** |
| 1. TDP-SP | **16** | **210** | 0 | 1753 | 0 | 1979 | **0.11** |
| 1. TDP-SP | **3** | **126** | 0 | 2565 | 0 | 2694 | **0.05** |
| 1. TDP-SP | **14** | **264** | 0 | 2068 | 0 | 2346 | **0.11** |
| 1. TDP-SP | **0** | **19** | 0 | 1515 | 0 | 1534 | **0.01** |
|  |  |  |  |  |  |  |  |
| 1. TDP-C9 | **10** | **219** | 0 | 2525 | 0 | 2754 | **0.08** |
| 1. TDP-C9 | **1** | **101** | 1 | 1542 | 0 | 1645 | **0.06** |
| 1. TDP-C9 | **31** | **261** | 2 | 1807 | 0 | 2101 | **0.14** |
| 1. TDP-C9 | **18** | **227** | 4 | 1910 | 0 | 2159 | **0.11** |
| 1. TDP-C9 | **14** | **192** | 4 | 3691 | 0 | 3901 | **0.05** |
| 1. TDP-C9 | **3** | **93** | 1 | 1441 | 0 | 1538 | **0.06** |
| 1. TDP-C9 | **0** | **158** | 0 | 1930 | 0 | 2088 | **0.08** |
| 1. TDP-C9 | **7** | **137** | 4 | 3335 | 0 | 3483 | **0.04** |
| 1. TDP-C9 | **7** | **110** | 3 | 3499 | 0 | 3619 | **0.03** |
| 1. TDP-C9 | **9** | **92** | 5 | 1407 | 0 | 1513 | **0.07** |
| 1. TDP-C9 | **7** | **114** | 2 | 1650 | 0 | 1773 | **0.07** |
| 1. TDP-C9 | **11** | **237** | 3 | 2062 | 0 | 2313 | **0.11** |
| 1. TDP-C9 | **13** | **82** | 5 | 2018 | 0 | 2118 | **0.04** |
| 1. TDP-C9 | **20** | **225** | 3 | 3521 | 0 | 3769 | **0.07** |
| 1. TDP-C9 | **5** | **102** | 2 | 2246 | 0 | 2355 | **0.05** |
| 1. TDP-C9 | **15** | **165** | 2 | 1669 | 0 | 1851 | **0.10** |
|  |  |  |  |  |  |  |  |
| **Case no. Neuropathological diagnosis** | **GABRQ Positive VENs** | **GABRQ Positive Pyr** | **GABRQ Negative VENs** | **GABRQ Negative Pyr** | **Unsure** | **All Neurons** | **GABRQ positive VENs & Pyr / total L5 neuron ratio** |
|  |  |  |  |  |  |  |  |
| 1. TDP-GRN | **5** | **139** | 1 | 1316 | 0 | 1461 | **0.10** |
| 1. TDP-GRN | **13** | **71** | 2 | 1434 | 0 | 1520 | **0.06** |
| 1. TDP-GRN | **6** | **253** | 0 | 2717 | 0 | 2976 | **0.09** |
| 1. TDP-GRN | **1** | **26** | 0 | 1563 | 0 | 1590 | **0.02** |
| 1. TDP-GRN | **0** | **45** | 0 | 1812 | 0 | 1857 | **0.02** |
| 1. TDP-GRN | **5** | **113** | 0 | 1240 | 0 | 1358 | **0.09** |
|  |  |  |  |  |  |  |  |
| **FTLD-Tau** |  |  |  |  |  |  |  |
| 1. Tau-SP | **5** | **136** | 0 | 2111 | 0 | 2252 | **0.06** |
| 1. Tau-SP | **8** | **266** | 1 | 1362 | 0 | 1637 | **0.17** |
| 1. Tau-SP | **7** | **322** | 0 | 2606 | 0 | 2935 | **0.11** |
| 1. Tau-SP | **52** | **432** | 1 | 3295 | 0 | 3780 | **0.13** |
|  |  |  |  |  |  |  |  |
| - - - 1. Tau-MAPT | **0** | **44** | 0 | 770 | 0 | 814 | **0.05** |
| - - - 1. Tau-MAPT | **6** | **59** | 2 | 394 | 0 | 461 | **0.14** |
| - - - 1. Tau-MAPT | **14** | **231** | 3 | 1836 | 0 | 2084 | **0.12** |
| - - - 1. Tau-MAPT | **50** | **310** | 25 | 2583 | 0 | 2968 | **0.12** |
| - - - 1. Tau-MAPT | **15** | **384** | 2 | 2837 | 0 | 3238 | **0.12** |
| - - - 1. Tau-MAPT | **4** | **343** | 0 | 3416 | 0 | 3763 | **0.09** |
| - - - 1. Tau-MAPT | **6** | **293** | 1 | 2006 | 0 | 2306 | **0.13** |
| - - - 1. Tau-MAPT | **1** | **53** | 1 | 339 | 0 | 394 | **0.14** |
| - - - 1. Tau-MAPT | **6** | **176** | 1 | 1852 | 0 | 2035 | **0.09** |
| - - - 1. Tau-MAPT | **5** | **149** | 1 | 1240 | 0 | 1395 | **0.11** |
|  |  |  |  |  |  |  |  |
| **FTLD-FUS** |  |  |  |  |  |  |  |
| 1. FUS | **0** | **21** | 0 | 1310 | 0 | 1331 | **0.02** |
| 1. FUS | **4** | **137** | 0 | 2168 | 0 | 2309 | **0.06** |
| 1. FUS | **0** | **96** | 0 | 1665 | 0 | 1761 | **0.05** |
| 1. FUS | **2** | **106** | 0 | 1555 | 0 | 1663 | **0.06** |
| 1. FUS | **4** | **162** | 1 | 3312 | 0 | 3479 | **0.05** |
| 1. FUS | **7** | **101** | 0 | 1421 | 0 | 1529 | **0.07** |
| 1. FUS | **2** | **29** | 0 | 1325 | 0 | 1356 | **0.02** |
| 1. FUS | **1** | **49** | 0 | 845 | 0 | 895 | **0.06** |
|  |  |  |  |  |  |  |  |
| **AD** |  |  |  |  |  |  |  |
| - - - 1. AD | **11** | **178** | 3 | 2081 | 0 | 2273 | **0.08** |
| - - - 1. AD | **54** | **453** | 4 | 2055 | 0 | 2566 | **0.20** |
| - - - 1. AD | **22** | **235** | 4 | 1525 | 0 | 1786 | **0.14** |
| - - - 1. AD | **53** | **510** | 2 | 5546 | 0 | 6111 | **0.09** |
| - - - 1. AD | **16** | **277** | 0 | 2267 | 0 | 2560 | **0.11** |
| - - - 1. AD | **11** | **179** | 0 | 1504 | 0 | 1694 | **0.11** |
| - - - 1. AD | **11** | **273** | 0 | 1135 | 0 | 1419 | **0.20** |
| VENs –Von Economo Neurons; Pyr – Pyramidal Neurons; Ratio – Ratio of GABRQ-expressing neurons (GABRQ/total L5-ratio); FTLD–frontotemporal lobar dementia; TDP-TAR DNA-binding protein 43; SP–sporadic; C9-C9orf72; GRN–progranulin; MAPT-Microtubule Associated Protein Tau; AD-Alzheimer’s disease. | | | | | | | |
